# Assessing educational disparities in COVID-19 related excess mortality in Spain: a population register-linked mortality study

**DOI:** 10.3389/fpubh.2024.1381298

**Published:** 2024-08-27

**Authors:** José Pulido, Marta Donat, Almudena Moreno, Julieta Politi, Lucía Cea-Soriano, Luis Sordo, Alberto Mateo-Urdiales, Elena Ronda, María José Belza, Gregorio Barrio, Enrique Regidor

**Affiliations:** ^1^Department of Public Health and Maternal and Child Health, Faculty of Medicine, Universidad Complutense de Madrid, Madrid, Spain; ^2^CIBER Epidemiología y Salud Pública (CIBERESP), Madrid, Spain; ^3^Department of Sociology, Universidad Pública de Navarra, Pamplona, Spain; ^4^Department of Infectious Diseases, Istituto Superiore di Sanità, Rome, Italy; ^5^Preventive Medicine and Public Health Area, Universidad de Alicante, Alicante, Spain; ^6^National School of Public Health, Instituto de Salud Carlos III, Madrid, Spain

**Keywords:** COVID-19 pandemic, mortality, cause of death, education, Spain

## Abstract

**Introduction:**

Data on the increase in mortality during the COVID-19 pandemic based on individuals' socioeconomic positions are limited. This study examines this increase in mortality in Spain during the epidemic waves of 2020 and 2021.

**Methods:**

We calculated the overall and cause-specific mortality rates during the 2017–2019 pre-pandemic period and four epidemic periods in 2020 and 2021 (first, second, third-fourth, and fifth-sixth waves). Mortality rates were analyzed based on educational levels (low, medium, and high) and across various age groups (25–64, 65–74, and 75+). The increase in mortality during each epidemic period compared to the pre-pandemic period was estimated using mortality rate ratios (MRR) derived from Poisson regression models.

**Results:**

An inverse educational gradient in overall mortality was observed across all periods; however, this pattern was not consistent for COVID-19 mortality in some age groups. Among those aged 75 years and older, highly educated individuals showed higher COVID-19 mortality during the first wave. In the 25–64 age group, individuals with low education experienced the highest overall mortality increase, while those with high education had the lowest increase. The MRRs were 1.21 and 1.06 during the first wave and 1.12 and 0.97 during the last epidemic period. In the 65–74 age group, highly educated individuals showed the highest overall mortality increase during the first wave, whereas medium-educated individuals had the highest increase during the subsequent epidemic periods. Among those aged 75 and older, highly educated individuals exhibited the highest overall mortality increase while the individuals with low education showed the lowest overall mortality increment, except during the last epidemic period.

**Conclusion:**

The varying educational patterns of COVID-19 mortality across different age groups contributed to the disparities of findings in increased overall mortality by education levels during the COVID-19 pandemic.

## Introduction

Numerous investigations have estimated mortality rates during the COVID-19 pandemic based on the socioeconomic status. Most studies indicate that COVID-19 mortality is highest among individuals with low socioeconomic status (1, 2). However, this evidence does not clarify whether the increase in overall mortality within each socioeconomic category, compared to the pre-pandemic period, has been greater among low socioeconomic groups.

Three studies conducted in the USA, South Korea, and California, along with two studies in Spain, are exceptions to the general findings (3–7). In the first two studies, carried out in USA and South Korea, individuals with low and high education showed the highest and lowest excess overall mortality during 2020 compared to the pre-pandemic period, respectively (3, 4). The analysis of mortality from leading causes of death in both studies revealed similar findings. Mortality from some causes such as cancer and heart disease increased in individuals with low education but decreased in those with high education. Mortality caused by respiratory diseases decreased overall, with a smaller decrease observed in individuals with low education compared to those with high education. The analysis the California population also analyzed overall mortality across different age groups, consistently finding the greatest excess mortality in 2020 among individuals with low education.

In one of the two studies conducted in Spain, the greatest decrease in life expectancy in 2020, compared to the pre-pandemic period, was observed among individuals with low education (6). The authors did not estimate life expectancy at different ages. According to the National Institute of Statistics of Spain (INE), life expectancy in 2020 at 65 years of age, compared to life expectancy in 2019, decreased by 1.2 years for individuals with low education and 1.4 years for those with high education (8). Between 25 and 64 years of age, the decrease in life expectancy was 0.5 years for individuals with low education and 0.1 years for those with high education. These findings suggest a greater increase in overall mortality rates during 2020 among highly educated older individuals, while the opposite was true for the working-age population, as confirmed in the other Spanish investigation (7).

A Spanish national seroprevalence study of anti-SARS-CoV-2 antibodies found that highly educated individuals had the highest incidence of SARS-CoV-2 infection during the first epidemic wave (9). The significant increase in overall mortality rates during 2020 among highly educated people, specifically those aged 65 years and older, could reflect an elevated COVID-19 mortality due to this higher infection incidence.

This seroprevalence study also found a higher incidence of SARS-CoV-2 infection among immigrants compared to the general Spanish population (9, 10). The excess overall mortality in individuals with low education aged 25–64 years may reflect high COVID-19 mortality among immigrants, as a significant proportion of poorly educated working-age individuals are immigrants (11).

The hypothesis of this study is that the variation in excess overall mortality by education is due to differences in the relationship between educational level and COVID-19 mortality by age. Understanding these changes in mortality patterns by educational level during the COVID-19 pandemic is crucial for public health practice and health policy in Spain and countries with similar epidemiological characteristics. The observed findings can guide public health responses in future epidemics caused by respiratory transmitted viruses such as SARS-CoV-2. Similarly, examining excess mortality from leading causes of death can inform interventions for vulnerable populations and the allocation of public health resources.

To understand the impact of the COVID-19 pandemic on mortality rates by education levels in Spain, this study calculated both the overall mortality and COVID-19-specific mortality rates across different age groups during the 2020 and 2021 COVID-19 epidemic waves. Similarly, it estimated changes in overall mortality and mortality from the leading causes of death for each educational level compared to the pre-pandemic period.

## Methods

Two data files covering the entire population residing in Spain during 1st January and 1st July of each year from 2017 to 2021 were provided by the INE. One file was stratified by 5-year age groups and educational levels, while the other was stratified by 5-year age groups and country of birth. The INE also provided individual death data files, with personal identification removed, for deaths of residents in Spain occurring between 1 January 2017, and 31 December 2021. These death data files included the age, country of birth, educational level of the deceased, and the cause, month, and year of death. All variables, except the educational level, were collected from the medical death certificate. The INE assigned the educational level to each deceased person aged 25 years and older based on information from the population registry. The underlying cause of death was coded according to the International Classification of Diseases, 10th revision.

The pre-pandemic period used to calculate excess mortality during the pandemic varies from one study to another. In this analysis, the selection of pre-pandemic and pandemic periods considered the availability of population and death data and the timing of the different epidemic waves in Spain. During 2020 and 2021, six epidemic waves of the COVID-19 pandemic were identified in Spain (12):

1. January 29 to June 21, 2020 (first wave)
2. June 22 to December 6, 2020 (second wave)
3. December 7, 2020, to March 14, 2021 (third wave)
4. March 15, 2021, to June 19, 2021 (fourth wave)
5. June 20, 2021, to October 13, 2021 (fifth wave)
6. October 14, 2021, to March 27, 2022 (sixth wave)

The analysis focused on deaths during four epidemic periods:

1. Deaths from the first half of 2020, as a proxy for deaths during the first wave.
2. Deaths from the second half of 2020, as a proxy for deaths during the second wave.
3. Deaths from the first half of 2021, as a proxy for deaths during the third and fourth waves.
4. Deaths from the second half of 2021, as a proxy for deaths during the fifth and sixth waves.

To estimate excess mortality during each epidemic period compared to pre-pandemic levels, mortality data from 2017, 2018, and 2019 were used as a reference. The average mortality during the first semester of those years served as the reference for the first and third epidemic periods, while the average mortality rate during the second semester was used as the reference for the second and fourth epidemic periods. All analyses were stratified into three broad age groups: 25–64 years, 65–74 years, and 75 years and older. The educational level was categorized as low (primary education or less –up to 6 years of education–), medium (secondary education –up to 12 years–), and high (bachelor's degree or higher). In Spain, the educational level is the only measure of socioeconomic position included in the annual files of population and deaths.

For this reason, this is the measure used in the present study.

A total of 1.9% of the deceased were excluded due to the absence of information on the education level in the death files. Supplementary Table S1 shows the number of deaths and population for each combination of broad age groups and educational levels. Supplementary Table S2 shows the number of deaths from COVID-19 by month and population on 1 January and 1 July, according to 5-year age groups and educational levels in 2020 and 2021.

In each broad age group, the age-standardized overall mortality rate per 100,000 population according to education was calculated for both the pre-pandemic period and the four epidemic periods. The age-standardized COVID-19 mortality rate per 100,000 population according to education was also calculated for the epidemic periods. The weights for standardization were derived from the 2013 European Standard Population. Graphs were used to present the overall mortality rates and COVID-19 mortality rates according to education in the analyzed periods.

For each educational level within each of the three large age groups, the relative excess in overall mortality during each epidemic period was estimated by calculating the age-adjusted mortality rate ratio (MRR) compared to the same pre-pandemic period. Additionally, the age-adjusted MRR was calculated for all causes of death other than COVID-19 and for each of the leading causes of death, including cancer, heart disease, cerebrovascular disease, Alzheimer's disease, chronic lower respiratory disease, unintentional injuries, diabetes, pneumonia and influenza, hypertensive disease, and kidney disease. Poisson regression models were used for these calculations, as shown below:


$$\begin{array}{l} D=\alpha +P+{\text{β}}_{1}X_{1}+{\text{β}}_{2}X_{2}+\Sigma {⋎}_{\text{i}}Z_{\text{i}}+e \end{array}$$

The number of deaths (D) was the outcome variable, and population (P) was included as an offset variable. Each model included two independent dummy variables corresponding to the epidemic periods (X_1_ and X_2_) and several independent dummy variables corresponding to the 5-year age categories (Z_i_). In the models that included deaths from the first half of 2017–2019, 2020, and 2021, the exp (β_1_) and exp (β_2_) represent the age-adjusted MRR for the first and third epidemic periods, respectively. In the models that included deaths from the second half of 2017–2019, 2020, and 2021, the exp (β_1_) and exp (β_2_) represent the age-adjusted MRR for the second and fourth epidemic periods, respectively.

Given that the prevalence of SARS-CoV-2 infection was higher among immigrants than in the Spanish population (9, 10), mortality from COVID-19 according to the country of birth was also calculated. This variable was grouped into the following six regions: Spain, the rest of Europe, Africa, Central America and the Caribbean, South America, and Asia. For each age group, the age-standardized mortality rate from COVID-19 per 100,000 population according to the region of birth was calculated for each epidemic period.

In this study, all data analyses were conducted using SAS software (v.9.4) for Windows.

## Results

First, two figures illustrate both the overall mortality and COVID-19-specific mortality based on education levels across different age groups during the COVID-19 epidemic waves of 2020 and 2021. Subsequently, tables with MRR are shown to reflect changes in overall mortality and mortality from the leading causes of death at each educational level during the epidemic waves compared to the pre-pandemic period. Finally, a table with MRR shows changes in mortality by region of birth.

**Figure 1:**
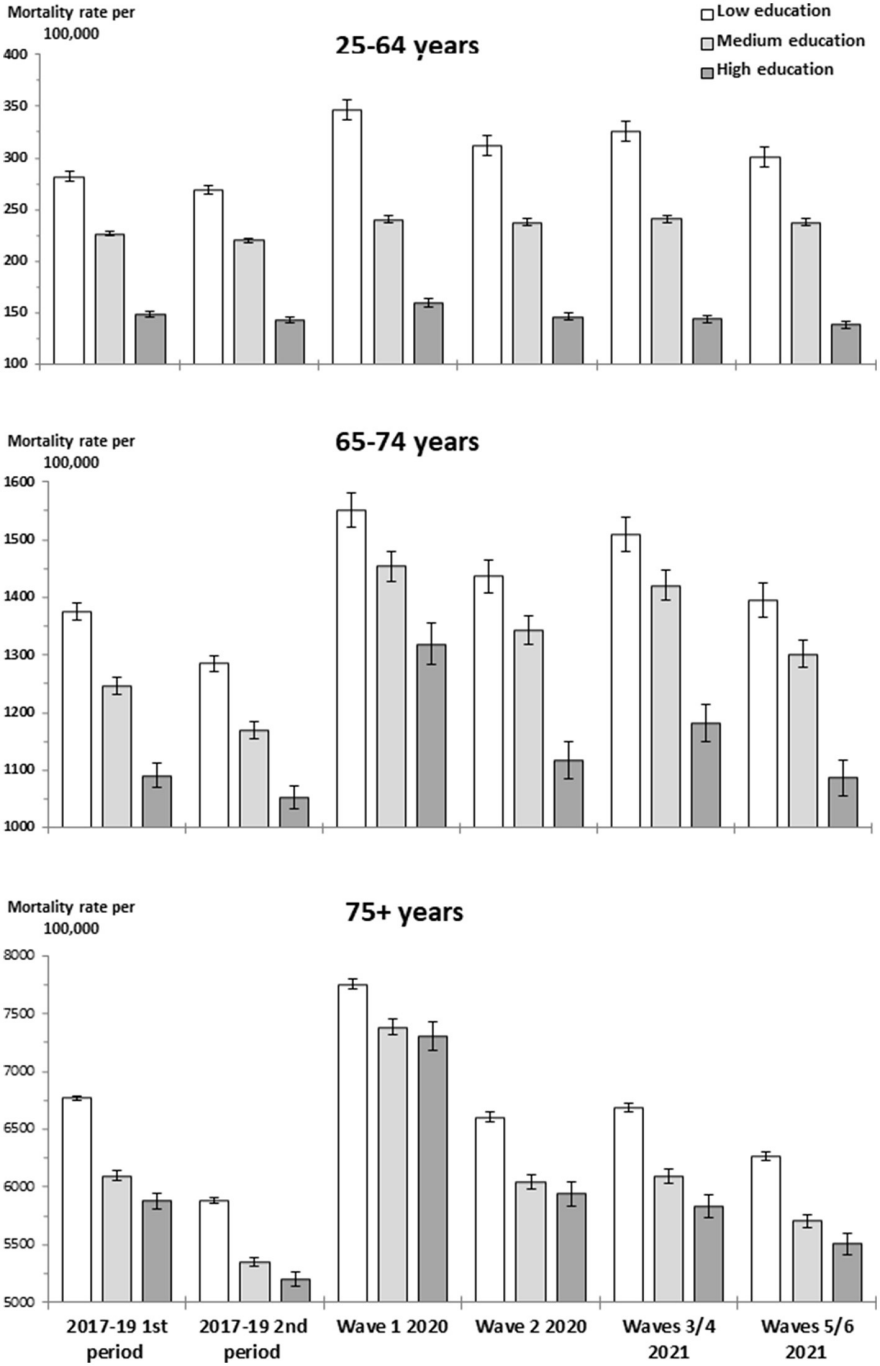
Age-standardized overall mortality rate by age group and educational level during the pre-pandemic periods and during the epidemic periods of 2020 and 2021.

Figure 1 shows an inverse educational gradient in overall mortality across all broad age groups and periods analyzed.

**Figure 2:**
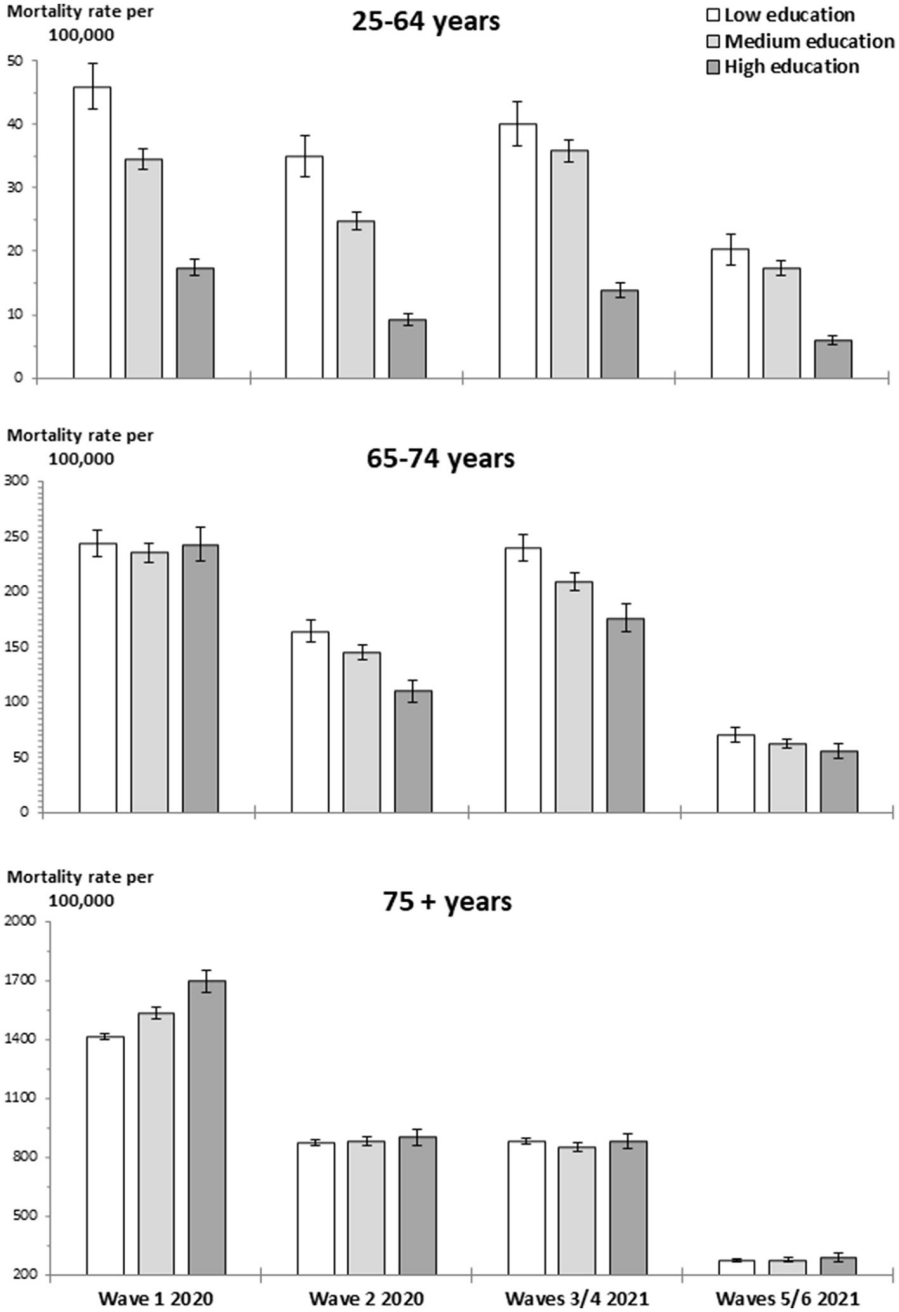
Age-standardized COVID-19 mortality rate by age group and educational level during the epidemic periods of 2020 and 2021. Deaths from COVID-19 correspond to codes U07.1 and U07.2 of the International Classification of Disease, 10th revision.

Figure 2 shows COVID-19 mortality based on education levels. For individuals under 75 years old, COVID-19 mortality showed an inverse educational gradient, except during the first wave for the 65–74 age group, where mortality was similar across educational levels. In those aged 75 years and older, COVID-19 mortality showed a direct educational gradient during the first wave. While in the remaining epidemic periods, the mortality rate was slightly higher among highly educated individuals compared to those with medium and low education levels. In individuals under the age of 75 years, COVID-19 mortality increased at all educational levels during the third and fourth waves compared to the previous epidemic period. However, in those aged 75 years and older, mortality remained similar in both epidemic periods.

Table 1 shows the overall MRR during the epidemic periods compared to the pre-pandemic period for each educational level. In the 25–64 age group, the MRR was the highest among those with low education and lowest among those with high education. Specifically, the MRR for poorly educated individuals was 1.21 (95% CI 1.17–1.25) during the first wave and 1.12 (1.08–1.16) during the fifth and sixth waves. The MRR was 1.06 (1.03–1.09) for highly educated individuals during the first wave and 0.97 (0.94–1.00) during the fifth and sixth waves.

**Table 1:** Mortality rate ratio and 95% confidence interval for overall mortality and for all causes other than COVID-19^a^.

**Age group and educational level**	**Pre-pandemic period (2017–2019)**	**1st wave 2020**	**2nd wave 2020**	**3rd and 4th waves 2021**	**5th and 6th waves 2021**
	**MRR**	**MRR (95% CI)**	**MRR (95% CI)**	**MRR (95% CI)**	**MRR 95% CI**
**OVERALL MORTALITY**					
**25–64 yrs**					
Low	1.00	1.21 (1.17, 1.25)	1.16 (1.12, 1.20)	1.16 (1.12, 1.20)	1.12 (1.08, 1.16)
Medium	1.00	1.06 (1.04, 1.08)	1.08 (1.06, 1.10)	1.06 (1.05, 1.08)	1.08 (1.06, 1.10)
High	1.00	1.06 (1.03, 1.09)	1.02 (0.99, 1.05)	0.97 (0.94, 1.00)	0.97 (0.94, 1.00)
**65–74 yrs**					
Low	1.00	1.13 (1.10, 1.15)	1.11 (1.09, 1.14)	1.09 (1.06, 1.11)	1.08 (1.06, 1.11)
Medium	1.00	1.17 (1.15, 1.19)	1.13 (1.11, 1.16)	1.13 (1.11, 1.15)	1.11 (1.09, 1.13)
High	1.00	1.21 (1.17, 1.25)	1.06 (1.02, 1.09)	1.08 (1.05, 1.12)	1.03 (1.00, 1.07)
≥**75 yrs**					
Low	1.00	1.14 (1.14, 1.15)	1.12 (1.12, 1.13)	0.98 (0.97, 0.99)	1.07 (1.06, 1.07)
Medium	1.00	1.21 (1.20, 1.22)	1.13 (1.12, 1.14)	1.00 (0.99, 1.02)	1.07 (1.05, 1.08)
High	1.00	1.24 (1.22, 1.27)	1.14 (1.12, 1.17)	1.00 (0.98, 1.02)	1.06 (1.04, 1.08)
**ALL CAUSES OTHER THAN COVID-19**					
**25–64 yrs**					
Low	1.00	1.05 (1.02, 1.09)	1.03 (0.99, 1.06)	1.02 (0.98, 1.05)	1.05 (1.01, 1.09)
Medium	1.00	0.97 (0.95, 0.98)	1.01 (0.99, 1.03)	0.97 (0.95, 0.98)	1.03 (1.01, 1.05)
High	1.00	0.95 (0.93, 0.98)	0.96 (0.93, 0.99)	0.88 (0.85, 0.91)	0.93 (0.90, 0.96)
**65–74 yrs**					
Low	1.00	0.95 (0.93, 0.97)	0.99 (0.97, 1.01)	0.91 (0.89, 0.93)	1.01 (0.99, 1.03)
Medium	1.00	0.98 (0.96, 1.00)	1.03 (1.01, 1.06)	0.96 (0.94, 0.98)	1.05 (1.03, 1.07)
High	1.00	0.99 (0.95, 1.02)	0.95 (0.92, 0.99)	0.92 (0.89, 0.96)	0.98 (0.95, 1.01)
≥**75 yrs**					
Low	1.00	0.94 (0.93, 0.94)	0.98 (0.97, 0.98)	0.86 (0.85, 0.86)	1.02 (1.01, 1.03)
Medium	1.00	0.96 (0.95, 0.97)	0.97 (0.95, 0.98)	0.86 (0.85, 0.87)	1.01 (1.00, 1.03)
High	1.00	0.95 (0.93, 0.98)	0.97 (0.95, 0.99)	0.85 (0.83, 0.86)	1.00 (0.98, 1.03)

^a^The reference for the first wave and for the third and fourth waves has been the average mortality rate during the first half of 2017–2019. The reference for the second and for the fifth and sixth waves has been the average mortality rate during the second half of 2017–2019.

In the 65–74 age group, all MRRs were statistically significant. The highest MRRs were observed in medium-educated individuals, while the lowest was in highly educated individuals, except during the first wave, where the highest MRR was observed in highly educated individuals and the lowest in poorly educated individuals.

In those aged 75 years and older, the highest MRRs were observed in highly educated individuals and the lowest in poorly educated individuals, except during the last epidemic period analyzed. Specifically, the MRR for poorly educated individuals was 1.14 (1.14–1.15) during the first wave and 1.07 (1.06–1.07) during the fifth and sixth waves. The MRR was 1.24 (1.2–1.27) for highly educated individuals during the first wave and 1.06 (1.06–1.08) during the fifth and sixth waves.

Table 1 also shows the MRR from all causes of death other than COVID-19 during the epidemic periods compared to the pre-pandemic periods for each educational level. Most MRRs were lower than 1.00, indicating a relative decrease in mortality compared to the pre-pandemic period. An exception was observed for poorly educated individuals in the 25–64 age group, where the MRRs ranged between 1.02 and 1.05 across different epidemic periods. However, only the MRRs during the first and fourth epidemic periods were statistically significant.

The lowest MRRs were observed during the third and fourth waves of the epidemic, mainly among those aged 75 years and older. In this age group, the MRRs for poorly educated, medium-educated, and highly educated individuals were 0.86 (0.85–0.86), 0.86 (0.85–0.87), and 0.85 (0.83–0.86), respectively.

Generally, mortality from the leading causes of death decreased during the various epidemic periods compared to the pre-pandemic period (Supplementary Tables S3–S5). Exceptions included increases in mortality from diabetes and hypertensive disease. Additionally, increases in mortality from heart disease and cerebrovascular disease were observed in individuals under 75 years of age and from unintentional injuries in the 25–64 age group. In most epidemic periods, the greatest increase in mortality from these causes compared to the pre-pandemic period was observed among poorly educated individuals.

Table 2 shows COVID-19 mortality by region of birth. For individuals under 75 years old, immigrants from South America, Africa, and Asia showed the highest mortality rates in most epidemic periods. In the 25–64 age group, immigrants from Central America and the Caribbean had higher mortality rates than Spanish individuals during the first and second waves. Among those aged 75 years and older, the highest mortality was observed in Spanish individuals, except during the last epidemic period analyzed. Immigrants from the rest of Europe had lower COVID-19 mortality than Spanish individuals across almost all epidemic periods and age groups.

**Table 2:** Mortality rate from COVID-19 per 100,000 population (95% confidence interval) by place of birth^a^.

**Age group and place of birth**	**1st wave 2020**	**2nd wave 2020**	**3rd and 4th waves 2021**	**5th and 6th waves 2021**
	**MR (95% CI)** ^b^	**MR (95% CI)** ^b^	**MR (95% CI)** ^b^	**MR (95% CI)** ^b^
**25–64 yrs**				
Spain	21.4 (20.6, 22.2)	13.9 (13.2, 14.6)	20.7 (19.8, 21.5)	8.5 (8.0, 9.1)
Rest of Europe	9.2 (7.0, 11.4)	10.3 (7.8, 12.8)	11.5 (9.1, 14.0)	16.7 (13.8, 19.6)
Africa	17.5 (12.4, 22.7)	32.5 (25.6, 39.3)	31.3 (24.6, 38.1)	15.5 (10.8, 20.2)
Central America & the Caribbean	25.0 (16.5, 33.6)	15.2 (8.2, 22.2)	17.5 (10.0, 25.0)	7.2 (2.3, 12.0)
South America	44.8 (39.3, 50.2)	30.3 (26.0, 34.7)	29.3 (25.2, 33.4)	15.3 (12.4, 18.3)
Asia	27.9 (16.9, 38.8)	22.4 (12.6, 32.3)	19.2 (10.2, 28.1)	19.6 (11.1, 28.2)
**65–74 yrs**				
Spain	241.8 (235.2, 248.4)	140.8 (135.8, 145.9)	209.9 (203.7, 216.0)	61.1 (57.7, 64.4)
Rest of Europe	54.4 (39.9, 68.9)	44.3 (30.5, 58.0)	118.9 (97.9, 139.9)	57.9 (43.3, 72.6)
Africa	176.9 (122.0, 231.8)	324.7 (252.0, 397.3)	247.4 (185.9, 308.9)	91.0 (54.5, 127.5)
Central America & the Caribbean	233.1 (146.0, 320.1)	119.5 (58.4, 180.5)	155.0 (88.1, 221.8)	77.2 (29.0, 125.4)
South America	359.8 (305.9, 413.7)	325.3 (273.7, 376.8)	358.7 (306.8, 410.7)	116.9 (88.2, 145.7)
Asia	212.9 (113.8, 312.0)	160.5 (75.4, 245.6)	276.1 (171.1, 381.0)	146.5 (69.2, 223.8)
≥**75 yrs**				
Spain	2,115.1 (2,092.9, 2,137.2)	1,268.8 (1,251.6, 1,285.9)	873.8 (861.8, 885.8)	276.9 (270.3, 283.5)
Rest of Europe	598.5 (519.9, 677.1)	481.0 (400.5, 561.5)	500.8 (445.4, 556.3)	196.8 (162.2, 231.3)
Africa	1,131.0 (915.1, 1,346.9)	1,222.8 (988.8, 1,456.8)	780.8 (634.0, 927.6)	521.9 (402.0, 641.9)
Central America & the Caribbean	817.2 (620.7, 1,013.7)	778.1 (570.3, 986.0)	390.6 (257.1, 524.1)	133.6 (54.6, 212.7)
South America	1,385.6 (1,213.8, 1,557.4)	1,138.3 (969.8, 1,306.9)	854.0 (738.0, 970.0)	380.7 (295.2, 466.1)
Asia	380.6 (163.7, 597.4)	605.3 (282.7, 927.9)	484.6 (270.3, 698.9)	202.5 (56.6, 348.5)

^a^People born in places other than those that appear in the table was excluded from the analysis. They represent 0.29% in the 25–64 age group, 0.14% in the 65–74 age group, and 0.07% in the 75 years and older group.

^b^95% confidence interval.

## Discussion

The COVID-19 pandemic imposed an additional burden by exacerbating chronic underlying conditions that can lead to death (13–15). Similarly, the presence of chronic conditions increased the lethality of COVID-19 patients, causing many individuals to die from COVID-19 rather than their pre-existing diseases. The potential for COVID-19 to trigger ischemic stroke and acute myocardial infarction cannot be ruled out (16–18). An increase in mortality from unintentional accidental injuries, excluding traffic accidents, was also observed in young adults during the pandemic (19–21).

The findings of this study reveal that the increase in mortality from heart disease, cerebrovascular disease, and unintentional injuries in the 25–64 age group as well as the increase in mortality from diabetes and hypertensive disease in all age groups were smaller than the decrease in mortality from other leading causes of death.

Consequently, mortality from all causes other than COVID-19 decreased during the epidemic periods compared to the pre-pandemic period across all age groups and almost all educational levels. This decrease indicates that the observed excess overall mortality was largely due to COVID-19 deaths. These COVID-19 deaths also explain the variability in overall mortality increases based on education levels.

However, previous studies in the USA and South Korea found that the greatest excess overall mortality during the pandemic, compared to the pre-pandemic period, was observed among poorly educated individuals (3–5). Additionally, previous research in the USA has shown an inverse educational gradient in COVID-19 mortality (3, 22–24). In contrast, this inverse gradient was not consistently observed across several epidemic periods and age groups in this study.

Educational differences in access to health services are less plausible as an explanation for COVID-19 mortality among those aged 75 years and older. After the first wave, when the impact of the pandemic was greatest, COVID-19 mortality was similar across all education levels. The Spanish universal healthcare system, along with the implementation of non-pharmaceutical interventions, likely helped mitigate disparities in COVID-19 mortality rates. In addition, more than 90% of individuals in each educational category received a COVID-19 vaccine (25).

However, limited access to healthcare among poorly educated individuals under 75 years of age cannot be ruled out. Poorer social and economic resources to access private services, when public services were overwhelmed due to excessive demand during the pandemic, could have contributed to higher COVID-19 mortality in these individuals.

Among working-age adults, the highest COVID-19 mortality in individuals with low education was primarily responsible for the largest overall mortality increase observed during any epidemic period. Many poorly educated individuals have jobs that do not allow remote work and require direct contact with others, increasing their risk of exposure to the SARS-CoV-2 virus. Nevertheless, other factors may have contributed to the observed inverse educational gradient in COVID-19 mortality.

Among working-age adults and during most epidemic periods, we found higher COVID-19 mortality among immigrants from Africa, Central America and the Caribbean, South America, and Asia compared to the Spanish population. Immigrants from these places comprise 42, 14, and 9% of the poorly educated, medium-educated, and highly educated groups, respectively, partly explaining the educational gradient in COVID-19 mortality. This high COVID-19 mortality among immigrants significantly contributed to the largest overall mortality increase observed in poorly educated individuals.

Household size could be a key factor in the high COVID-19 mortality among immigrants. Compared to the Spanish population, immigrants have a higher percentage of households with three or more members (11). Studies have shown that households have the highest transmission rates of SARS-CoV-2, and exposure to family contacts significantly increases the transmission potential of the virus (26–28).

In Spain and other southern European countries, highly educated and/or high-income individuals showed the highest frequency of SARS-CoV-2 infection during the first wave (9, 29, 30). This finding has been attributed to the greater international mobility of high socioeconomic individuals for work or leisure. However, the results on COVID-19 mortality among working-age individuals do not support this explanation.

During the first wave, COVID-19 mortality in the 65–74 age group was similar among individuals with low and high education. This is likely due to within-household transmission of SARS-CoV-2 (31, 32). Susceptibility to infection increased sharply with age and was higher among spouses compared to other family contacts (26, 27). In Spain, two-thirds of this age group live with their partner, and most share a similar educational level (11). Thus, SARS-CoV-2 infection of either a poorly or highly educated individual could have resulted in similar COVID-19 deaths through within-household transmission.

Nonetheless, the greatest overall mortality increase compared to the pre-pandemic period was observed in highly educated individuals. This is because mortality from all causes other than COVID-19 did not change among individuals with high education but decreased among those with low education. The contribution of mortality among immigrants to this finding was probably small, since foreign-born population in this age group was similar in size for low and highly educated individuals within this age category.

In subsequent waves, citizens' reactive behavior to avoid contact with older relatives reduced the penetration of the virus into these individuals' households. Poorly educated individuals show the highest prevalence of comorbidities, while highly educated individuals show the lowest. Since many COVID-19 deaths occurred in people with pre-existing chronic diseases, individuals aged 65–74 years exhibited an inverse educational gradient in COVID-19 mortality. Furthermore, deaths from all causes other than COVID-19 contributed to the greatest overall mortality increase in medium-educated individuals.

Co-residence patterns could have also influenced the frequency of infection based on education levels in older age groups (31, 32). The life-course inverse educational gradient in mortality increases the risk of being widowed among poorly educated individuals older than 74 years. After this age, 44% of poorly educated individuals lived with their partner, compared to 57% of highly educated individuals (11), with most of them sharing similar educational levels. Therefore, during the first wave, SARS-CoV-2 infection was less likely to cause COVID-19 deaths through within-household transmission in poorly educated individuals than in highly educated individuals. Consequently, the overall mortality increase was greater among highly educated individuals.

After the first wave, COVID-19 mortality in this age group decreased and became very similar across all education categories. This could be explained by the absence of variation in the incidence of SARS-CoV-2 infection based on educational levels, likely due to decreased contact with family members and other non-pharmaceutical interventions. Additionally, the reduction in COVID-19 mortality could be attributed to vaccination efforts and the selective survival of the healthiest subjects at an advanced age.

## Strengths and weaknesses of the study

This is the first study to investigate COVID-19 mortality and the impact of the pandemic on mortality from all specific causes, according to individuals' socioeconomic status, during the epidemic waves of 2020 and 2021. Most previous studies focus only on the year 2020, and only one of those studies has provided results for different age groups.

Other socioeconomic factors and comorbidities not considered in this study could have confounded the results regarding education and COVID-19 mortality, especially if these factors are unevenly distributed across educational levels. Additionally, the selection of epidemic periods might not fully reflect the diverse health policy responses implemented during the pandemic.

In some cases, COVID-19 may not be the underlying cause of death in individuals who passed away from this disease and were certified as COVID-19 deaths. Conversely, some individuals who died from COVID-19 may have had another underlying cause of death recorded, mainly in the early stages of the pandemic due to a lack of diagnostic tests. However, such misclassification does not affect the findings on variation in overall mortality during each epidemic period across educational levels compared to the pre-pandemic period. Additionally, in the early stages of the pandemic, highly educated individuals could have had greater access to diagnostic tests, which could have affected the observed relationship between education and COVID-19 mortality.

In the present study, excess deaths that can be attributed to increased temperature have not been estimated. This omission primarily affects the results in people aged 75 years and older, as most excess deaths associated with high temperature levels are concentrated in this age group. The summer of 2020 was particularly warm in Spain. Therefore, part of the excess mortality rates observed during the second wave must be attributed to the high temperatures in the summer of 2020. According to the MoMo Report for that period, mortality attributable to excess temperatures was higher than that observed in the summers of 2017 and 2018 (33). In contrast, the impact of temperature on excess mortality during the summer of 2021 was probably small. A recent study showed no correlation between non-COVID deaths and temperature in 2021 (34). According to the MoMo report on mortality attributable to excess temperature in the summer of 2021 in Spain, only 1.2% of excess deaths can be attributed to the increase in temperature (35).

The influence of the circulation of viruses other than SARS-CoV-2 on the results was likely minimal. The circulation of the flu virus and other respiratory viruses during the 2020/2021 season was very low, which was due to the public health measures implemented to reduce the transmission of SARS-CoV-2 (36). However, during November–December 2021, a wave of flu virus activity was detected, but its severity was low compared to the three flu seasons prior to the COVID-19 pandemic (37).

## Conclusion

In Spain, an inverse educational gradient in overall mortality was found during any epidemic period in 2020 and 2021, but this was not consistently observed for COVID-19 mortality across some age groups. Among those aged 75 and older, highly educated individuals showed higher COVID-19 mortality during the first wave. In general, the greatest excess overall mortality compared to the pre-pandemic period was observed in poorly educated individuals aged 25–64 years, medium-educated individuals aged 65–74 years, and highly educated individuals aged 75 years and older.

The household size and co-residence patterns in Spain could have influenced the frequency of SARV-CoV-2 infection by educational level, leading to different patterns across all age groups. Consequently, COVID-19 mortality and excess overall mortality based on education levels during the pandemic differ from those observed in previous studies. These findings can guide public health responses to epidemics caused by respiratory viruses and encourage similar research in countries with epidemiological characteristics similar to those of Spain.

## Data Availability

The datasets presented in this study can be found in online repositories. The names of the repository/repositories and accession number(s) can be found in the article/Supplementary material.
