# Supplementary material for: Assessing educational disparities in COVID-19 related excess mortality in Spain: a population register-linked mortality study
Source: Front Public Health. 2024 Aug 27;12:1381298. doi: 10.3389/fpubh.2024.1381298 (PMC11384991; doi:10.3389/fpubh.2024.1381298)
Supplement: Supplementary file 1 [file Table_1.docx]

Appendix table 1. Number of deaths and population during the pre-pandemic periods and during the epidemic periods of COVID-19, by age and educational group.

	Pre-pandemic (2017-19 1st period)	Pre-pandemic (2017-19 2nd period)	1st wave 2020	2nd wave 2020	3rd & 4th waves 2021	5th & 6th waves 2021
**All deaths[1]**						
**25-64 yrs**						
Low	5,355	4,979	5,054	4,521	4,594	4,138
Medium	10,745	10,458	12,114	12,049	12,032	11,986
High	5,558	5,521	6,657	6,336	6,474	6,387
**65-74 yrs**						
Low	12,239	11,117	11,422	10,134	10,069	9,056
Medium	8,917	8,572	12,210	11,381	12,080	11,247
High	3,836	3,839	5,371	4,731	5,252	4,985
**≥75 yrs**						
Low	121,031	104,476	137,000	114,497	113,829	106,967
Medium	20,733	19,011	30,742	26,223	27,034	25,836
High	10,118	9,278	14,586	12,346	12,880	12,481
**Population (in thousands)[2]**						
**25-64 yrs**						
Low	3,058.4	3,008.2	2,337.7	2,321.1	2,270.2	2,243.7
Medium	7,842.4	7,789.4	8,061.8	7,936.0	7,797.2	7,736.0
High	9,602.9	9,709.8	10,213.3	10,356.6	10,630.7	10,670.9
**65-74 yrs**						
Low	1,722.3	1,677.3	1,409.9	1,361.9	1,293.6	1,252.3
Medium	1,460.1	1,493.4	1,700.0	1,712.2	1,719.0	1,745.4
High	727.7	753.4	841.4	875.4	916.7	945.9
**≥75 yrs**						
Low	3,031.6	2,998.9	2,844.9	2,906.9	2,755.6	2,788.3
Medium	725.2	759.0	948.4	904.3	1,010.2	972.9
High	364.8	379.6	450.7	431.8	494.1	477.9

1. Deaths during the pre-pandemic periods correspond to the average of the three years (2017-2019).
2. Population for the 1st pre-pandemic period, 1st wave 2020 and 3rd & 4th waves 2021 refer to the population on January 1 and population for the 2nd pre-pandemic period, 2nd wave 2020, and 5th & 6th waves 2021 refer to the population on July 1, respectively. In the case of the years 2017-2019, it corresponds to the average population on those dates of the three years.
