# Supplementary material for: Assessing educational disparities in COVID-19 related excess mortality in Spain: a population register-linked mortality study
Source: Front Public Health. 2024 Aug 27;12:1381298. doi: 10.3389/fpubh.2024.1381298 (PMC11384991; doi:10.3389/fpubh.2024.1381298)
Supplement: Supplementary file 2 [file Table_3.docx]

Appendix table 3. Mortality rate ratio and 95% confidence interval for the leading causes of death[a].

Cause (ICD-10 code[b]), age group and educational level	Pre-pandemic period[1] (2017-2019)	1st wave 2020		2nd wave 2020		3rd & 4th waves 2021		5th & 6th waves 2021	
	Rate ratio	Rate ratio	95% CI	Rate ratio	95% CI	Rate ratio	95% CI	Rate ratio	95% CI
**CANCER (C00-C97)**									
**25-64 yrs**									
Low	1.00	0.99	(0.94 , 1.05)	0.95	(0.90 , 1.01)	0.97	(0.92 , 1.03)	0.93	(0.88 , 0.99)
Medium	1.00	0.95	(0.92 , 0.97)	0.95	(0.93 , 0.98)	0.92	(0.90 , 0.95)	0.96	(0.94 , 0.99)
High	1.00	0.94	(0.90 , 0.98)	0.89	(0.86 , 0.93)	0.87	(0.84 , 0.91)	0.87	(0.84 , 0.91)
**65-74 yrs**									
Low	1.00	0.92	(0.89 , 0.95)	0.95	(0.91 , 0.98)	0.91	(0.88 , 0.94)	0.96	(0.92 , 0.99)
Medium	1.00	0.98	(0.95 , 1.00)	1.00	(0.97 , 1.03)	0.98	(0.95 , 1.01)	1.03	(1.00 , 1.06)
High	1.00	1.00	(0.95 , 1.05)	0.95	(0.90 , 1.00)	0.98	(0.94 , 1.03)	0.95	(0.90 , 0.99)
**≥75 yrs**									
Low	1.00	0.97	(0.95 , 0.99)	0.97	(0.95 , 0.98)	0.97	(0.95 , 0.98)	1.01	(0.99 , 1.03)
Medium	1.00	0.98	(0.95 , 1.00)	0.98	(0.95 , 1.00)	0.95	(0.92 , 0.97)	1.01	(0.99 , 1.04)
High	1.00	0.96	(0.92 , 1.01)	0.99	(0.95 , 1.04)	0.93	(0.89 , 0.97)	1.01	(0.97 , 1.05)
**HEART DISEASE (I00-I09, I20-I51. I11-I13)**									
**25-64 yrs**									
Low	1.00	1.04	(0.95 , 1.14)	1.00	(0.98 , 1.01)	1.07	(0.98 , 1.17)	1.04	(1.02 , 1.05)
Medium	1.00	0.99	(0.94 , 1.03)	0.99	(0.96 , 1.02)	1.08	(1.04 , 1.13)	1.01	(0.99 , 1.04)
High	1.00	1.07	(0.98 , 1.16)	0.97	(0.92 , 1.02)	1.01	(0.93 , 1.10)	0.97	(0.93 , 1.02)
**65-74 yrs**									
Low	1.00	0.97	(0.92 , 1.03)	1.03	(0.97 , 1.10)	0.93	(0.88 , 0.99)	1.11	(1.04 , 1.17)
Medium	1.00	0.95	(0.90 , 1.00)	1.07	(1.01 , 1.12)	0.98	(0.93 , 1.03)	1.08	(1.02 , 1.13)
High	1.00	0.99	(0.91 , 1.09)	0.96	(0.87 , 1.05)	0.94	(0.86 , 1.03)	0.97	(0.89 , 1.07)
**≥75 yrs**									
Low	1.00	0.91	(0.90 , 0.93)	1.00	(0.98 , 1.01)	0.89	(0.88 , 0.91)	1.04	(1.02 , 1.05)
Medium	1.00	0.93	(0.90 , 0.95)	0.99	(0.96 , 1.02)	0.90	(0.88 , 0.93)	1.01	(0.99 , 1.04)
High	1.00	0.95	(0.91 , 1.00)	0.97	(0.92 , 1.02)	0.87	(0.83 , 0.91)	0.97	(0.93 , 1.02)
**CEREBROVASCULAR DISEASE (I60-I69)**									
**25-64 yrs**									
Low	1.00	1.04	(0.87 , 1.24)	1.16	(0.97 , 1.37)	1.13	(0.95 , 1.34)	1.10	(0.92 , 1.32)
Medium	1.00	1.01	(0.92 , 1.10)	1.05	(0.96 , 1.15)	1.02	(0.93 , 1.12)	0.96	(0.87 , 1.06)
High	1.00	0.88	(0.74 , 1.04)	1.04	(0.89 , 1.23)	0.83	(0.70 , 0.98)	0.90	(0.77 , 1.07)
**65-74 yrs**									
Low	1.00	0.90	(0.81 , 0.99)	1.12	(1.01 , 1.23)	1.08	(0.98 , 1.20)	1.04	(0.93 , 1.15)
Medium	1.00	0.94	(0.86 , 1.03)	1.08	(0.99 , 1.18)	1.01	(0.92 , 1.10)	1.00	(0.91 , 1.09)
High	1.00	0.78	(0.65 , 0.94)	1.09	(0.92 , 1.29)	0.83	(0.70 , 0.98)	1.05	(0.88 , 1.24)
**≥75 yrs**									
Low	1.00	0.94	(0.91 , 0.96)	0.95	(0.93 , 0.98)	0.87	(0.84 , 0.89)	0.95	(0.92 , 0.97)
Medium	1.00	0.97	(0.92 , 1.02)	0.95	(0.90 , 0.99)	0.89	(0.85 , 0.94)	0.97	(0.92 , 1.01)
High	1.00	0.98	(0.90 , 1.07)	0.91	(0.83 , 0.99)	0.83	(0.75 , 0.90)	0.97	(0.89 , 1.05)
**ALZHEIMER DISEASE (G30)**									
**25-64 yrs**									
Low	1.00	1.11	(0.61 , 2.01)	0.84	(0.44 , 1.62)	0.92	(0.48 , 1.78)	1.06	(0.58 , 1.96)
Medium	1.00	0.99	(0.69 , 1.44)	1.15	(0.78 , 1.69)	0.93	(0.63 , 1.35)	0.97	(0.65 , 1.46)
High	1.00	1.71	(0.82 , 3.55)	1.40	(0.79 , 2.49)	1.05	(0.46 , 2.42)	0.73	(0.36 , 1.48)
**65-74 yrs**									
Low	1.00	0.94	(0.78 , 1.13)	0.92	(0.76 , 1.12)	0.90	(0.74 , 1.10)	0.98	(0.81 , 1.19)
Medium	1.00	1.16	(0.97 , 1.37)	1.10	(0.93 , 1.30)	0.95	(0.80 , 1.14)	0.96	(0.81 , 1.15)
High	1.00	1.08	(0.78 , 1.48)	1.13	(0.82 , 1.57)	0.88	(0.63 , 1.23)	1.11	(0.81 , 1.53)
**≥75 yrs**									
Low	1.00	1.01	(0.98 , 1.04)	0.97	(0.94 , 1.00)	0.77	(0.75 , 0.80)	0.92	(0.89 , 0.95)
Medium	1.00	1.08	(1.02 , 1.15)	1.04	(0.98 , 1.11)	0.76	(0.71 , 0.81)	0.94	(0.89 , 1.00)
High	1.00	1.14	(1.03 , 1.27)	1.02	(0.91 , 1.14)	0.76	(0.68 , 0.85)	0.97	(0.87 , 1.08)

a. The reference for the first wave and for the third and fourth waves has been the average mortality rate during the first half of the years 2017-2019. The reference for the second and for the fifth and sixth waves has been the average mortality rate during the second half of the years 2017-2019.
