# Supplementary material for: Assessing educational disparities in COVID-19 related excess mortality in Spain: a population register-linked mortality study
Source: Front Public Health. 2024 Aug 27;12:1381298. doi: 10.3389/fpubh.2024.1381298 (PMC11384991; doi:10.3389/fpubh.2024.1381298)
Supplement: Supplementary file 3 [file Table_4.docx]

Appendix table 4. Mortality rate ratio and 95% confidence interval for the leading causes of death[a].

Cause (ICD-10 code[b]), age group and educational level	Pre-pandemic period[1] (2017-2019)	1st wave 2020		2nd wave 2020		3rd & 4th waves 2021		5th & 6th waves 2021	
	Rate ratio	Rate ratio	95% CI	Rate ratio	95% CI	Rate ratio	95% CI	Rat e	95% CI
**CHRONIC LOWER RESPIRATORY DISEASE (J40-J47)**									
**25-64 yrs**									
Low	1.00	1.04	(0.84 , 1.27)	1.04	(0.82 , 1.33)	0.90	(0.72 , 1.12)	1.17	(0.93 , 1.48)
Medium	1.00	0.95	(0.84 , 1.08)	0.98	(0.84 , 1.13)	0.77	(0.67 , 0.89)	1.23	(1.08 , 1.42)
High	1.00	1.00	(0.75 , 1.35)	0.98	(0.72 , 1.36)	0.66	(0.47 , 0.92)	0.99	(0.73 , 1.36)
**65-74 yrs**									
Low	1.00	0.83	(0.75 , 0.93)	0.82	(0.72 , 0.93)	0.59	(0.52 , 0.67)	1.02	(0.90 , 1.15)
Medium	1.00	0.90	(0.82 , 0.99)	0.93	(0.83 , 1.04)	0.68	(0.61 , 0.75)	1.06	(0.95 , 1.18)
High	1.00	0.89	(0.73 , 1.10)	0.94	(0.76 , 1.17)	0.71	(0.57 , 0.88)	0.84	(0.67 , 1.05)
**≥75 yrs**									
Low	1.00	0.84	(0.81 , 0.87)	0.80	(0.77 , 0.84)	0.59	(0.57 , 0.61)	0.93	(0.89 , 0.97)
Medium	1.00	0.85	(0.80 , 0.91)	0.90	(0.84 , 0.97)	0.63	(0.59 , 0.67)	0.94	(0.88 , 1.01)
High	1.00	0.83	(0.74 , 0.93)	0.94	(0.83 , 1.07)	0.60	(0.53 , 0.68)	0.84	(0.74 , 0.96)
**UNINTENTIONAL INJURY (V01-X59, Y85-Y86)**									
**25-64 yrs**									
Low	1.00	1.25	(1.07 , 1.45)	1.22	(1.05 , 1.42)	1.30	(1.12 , 1.52)	1.20	(1.03 , 1.40)
Medium	1.00	1.09	(1.01 , 1.17)	1.12	(1.05 , 1.20)	1.15	(1.07 , 1.23)	1.13	(1.05 , 1.21)
High	1.00	0.93	(0.81 , 1.07)	1.02	(0.90 , 1.15)	0.98	(0.86 , 1.13)	1.01	(0.89 , 1.14)
**65-74 yrs**									
Low	1.00	0.96	(0.82 , 1.13)	0.97	(0.84 , 1.13)	0.98	(0.84 , 1.16)	1.06	(0.91 , 1.23)
Medium	1.00	0.87	(0.76 , 1.01)	1.07	(0.94 , 1.23)	0.99	(0.86 , 1.13)	1.12	(0.99 , 1.27)
High	1.00	0.93	(0.72 , 1.21)	0.88	(0.68 , 1.13)	0.95	(0.73 , 1.22)	0.87	(0.68 , 1.12)
**≥75 yrs**									
Low	1.00	0.97	(0.92 , 1.01)	0.99	(0.95 , 1.04)	0.96	(0.91 , 1.00)	1.06	(1.01 , 1.11)
Medium	1.00	0.87	(0.80 , 0.95)	0.91	(0.83 , 0.99)	0.94	(0.86 , 1.02)	1.00	(0.92 , 1.08)
High	1.00	0.97	(0.83 , 1.12)	0.95	(0.82 , 1.10)	1.04	(0.90 , 1.19)	1.25	(1.09 , 1.42)
**DIABETES (E10-E14)**									
**25-64 yrs**									
Low	1.00	0.94	(0.71 , 1.25)	1.48	(1.14 , 1.92)	1.21	(0.93 , 1.57)	1.26	(0.95 , 1.68)
Medium	1.00	0.97	(0.82 , 1.15)	1.32	(1.11 , 1.55)	1.04	(0.88 , 1.23)	1.26	(1.06 , 1.49)
High	1.00	1.08	(0.76 , 1.52)	0.97	(0.65 , 1.46)	0.84	(0.58 , 1.22)	1.23	(0.85 , 1.77)
**65-74 yrs**									
Low	1.00	1.13	(0.99 , 1.30)	1.08	(0.93 , 1.26)	1.17	(1.01 , 1.35)	1.07	(0.92 , 1.25)
Medium	1.00	1.17	(1.02 , 1.34)	1.12	(0.97 , 1.29)	1.14	(0.99 , 1.31)	1.09	(0.94 , 1.26)
High	1.00	1.12	(0.84 , 1.48)	1.55	(1.15 , 2.10)	1.01	(0.76 , 1.35)	1.25	(0.91 , 1.71)
**≥75 yrs**									
Low	1.00	1.07	(1.03 , 1.12)	1.16	(1.11 , 1.21)	1.04	(1.00 , 1.08)	1.16	(1.11 , 1.20)
Medium	1.00	1.23	(1.14 , 1.33)	1.18	(1.09 , 1.28)	1.05	(0.97 , 1.14)	1.13	(1.04 , 1.22)
High	1.00	1.13	(0.98 , 1.32)	1.26	(1.08 , 1.46)	0.85	(0.72 , 1.00)	0.98	(0.84 , 1.14)

a. The reference for the first wave and for the third and fourth waves has been the average mortality rate during the first half of the years 2017-2019. The reference for the second and for the fifth and sixth waves has been the average mortality rate during the second half of the years 2017-2019.

b. ICD-10. International Classification of Disease, 10th revision
