# Supplementary material for: Assessing educational disparities in COVID-19 related excess mortality in Spain: a population register-linked mortality study
Source: Front Public Health. 2024 Aug 27;12:1381298. doi: 10.3389/fpubh.2024.1381298 (PMC11384991; doi:10.3389/fpubh.2024.1381298)
Supplement: Supplementary file 4 [file Table_5.docx]

Appendix table 5. Mortality rate ratio and 95% confidence interval for the leading causes of death[a].

Cause (ICD-10 code[b]), age group and educational level	Pre-pandemic period (2017-2019)	1st wave 2020		2nd wave 2020		3rd & 4th waves 2021		5th & 6th waves 2021	
	MRR	MRR	95% CI	MRR	95% CI	MRR	95% CI	MRR	95% CI
**PNEUMONIA AND INFLUENZA (J10-J99)**									
**25-64 yrs**									
Low	1.00	1.11	(0.61 , 2.01)	0.84	(0.44 , 1.62)	0.92	(0.48 , 1.78)	1.06	(0.58 , 1.96)
Medium	1.00	0.99	(0.69 , 1.44)	1.15	(0.78 , 1.69)	0.93	(0.63 , 1.35)	0.97	(0.65 , 1.46)
High	1.00	1.71	(0.82 , 3.55)	1.40	(0.79 , 2.49)	1.05	(0.46 , 2.42)	0.73	(0.36 , 1.48)
**65-74 yrs**									
Low	1.00	0.94	(0.78 , 1.13)	0.92	(0.76 , 1.12)	0.90	(0.74 , 1.10)	0.98	(0.81 , 1.19)
Medium	1.00	1.16	(0.97 , 1.37)	1.10	(0.93 , 1.30)	0.95	(0.80 , 1.14)	0.96	(0.81 , 1.15)
High	1.00	1.08	(0.78 , 1.48)	1.13	(0.82 , 1.57)	0.88	(0.63 , 1.23)	1.11	(0.81 , 1.53)
**≥75 yrs**									
Low	1.00	1.01	(0.98 , 1.04)	0.97	(0.94 , 1.00)	0.77	(0.75 , 0.80)	0.92	(0.89 , 0.95)
Medium	1.00	1.08	(1.02 , 1.15)	1.04	(0.98 , 1.11)	0.76	(0.71 , 0.81)	0.94	(0.89 , 1.00)
High	1.00	1.14	(1.03 , 1.27)	1.02	(0.91 , 1.14)	0.76	(0.68 , 0.85)	0.97	(0.87 , 1.08)
**HYPERTENSIVE DISEASE (I10, I12,I15)**									
**25-64 yrs**									
Low	1.00	2.38	(1.46 , 3.87)	0.87	(0.48 , 1.59)	2.13	(1.27 , 3.56)	1.13	(0.65 , 1.98)
Medium	1.00	1.88	(1.38 , 2.55)	1.24	(0.88 , 1.77)	1.62	(1.17 , 2.22)	1.18	(0.82 , 1.68)
High	1.00	1.12	(0.61 , 2.05)	1.79	(0.98 , 3.27)	0.76	(0.39 , 1.50)	0.93	(0.45 , 1.93)
**65-74 yrs**									
Low	1.00	1.35	(1.03 , 1.76)	1.37	(1.02 , 1.83)	1.35	(1.03 , 1.79)	1.28	(0.94 , 1.74)
Medium	1.00	1.22	(0.93 , 1.60)	0.80	(0.47 , 1.35)	1.32	(1.02 , 1.72)	0.78	(0.47 , 1.31)
High	1.00	1.60	(0.98 , 2.61)	0.80	(0.47 , 1.35)	1.41	(0.86 , 2.32)	0.78	(0.47 , 1.31)
**≥75 yrs**									
Low	1.00	1.20	(1.14 , 1.26)	1.30	(1.24 , 1.37)	1.16	(1.10 , 1.22)	1.29	(1.23 , 1.36)
Medium	1.00	1.16	(1.05 , 1.29)	1.18	(1.06 , 1.31)	1.04	(0.94 , 1.15)	1.23	(1.11 , 1.37)
High	1.00	1.03	(0.86 , 1.25)	0.94	(0.77 , 1.15)	0.99	(0.82 , 1.18)	1.06	(0.87 , 1.27)
**KIDNEY DISEASE (N00-N07, N17-N19, N25-N27)**									
**25-64 yrs**									
Low	1.00	1.05	(0.70 , 1.57)	1.33	(0.88 , 2.00)	0.96	(0.62 , 1.47)	1.14	(0.73 , 1.77)
Medium	1.00	0.99	(0.73 , 1.34)	1.26	(0.94 , 1.68)	1.06	(0.79 , 1.43)	1.17	(0.87 , 1.57)
High	1.00	0.43	(0.19 , 0.97)	0.76	(0.40 , 1.45)	0.69	(0.36 , 1.31)	0.95	(0.54 , 1.70)
**65-74 yrs**									
Low	1.00	0.86	(0.69 , 1.08)	1.05	(0.84 , 1.32)	0.88	(0.70 , 1.11)	1.10	(0.87 , 1.39)
Medium	1.00	1.10	(0.90 , 1.34)	1.19	(0.96 , 1.48)	0.90	(0.73 , 1.11)	1.04	(0.83 , 1.31)
High	1.00	1.32	(0.91 , 1.91)	0.96	(0.63 , 1.47)	0.87	(0.57 , 1.32)	1.10	(0.75 , 1.63)
**≥75 yrs**									
Low	1.00	1.02	(0.98 , 1.07)	1.07	(1.02 , 1.12)	1.01	(0.96 , 1.06)	1.14	(1.09 , 1.20)
Medium	1.00	0.96	(0.88 , 1.05)	0.96	(0.87 , 1.06)	0.92	(0.84 , 1.00)	1.16	(1.07 , 1.27)
High	1.00	0.92	(0.78 , 1.07)	0.88	(0.75 , 1.04)	0.97	(0.83 , 1.12)	1.01	(0.87 , 1.18)

a. The reference for the first wave and for the third and fourth waves has been the average mortality rate during the first half of the years 2017-2019. The reference for the second and for the fifth and sixth waves has been the average mortality rate during the second half of the years 2017-2019.

b. ICD-10. International Classification of Disease, 10th revision
